# Are There More Lunatics Than Formerly?

**Published:** 1887-07-02

**Authors:** Hack Tuke


### ARE THERE MORE LUNATICS NOW THAN FORMERLY?

By Dr. Hack Tuke.

"In your 'Annotations' of June 18, you ask, *à propos* of the new lunatic asylum for Middlesex, 'What is the meaning of the terrible increase of lunacy among the pauper population?' Will you allow me to say, in reply, that the fact itself is not proved? On the contrary, the alleged increase is far more apparent than real. With the care now taken of the insane there must be an enormous accumulation of cases. This and other causes will account for an increase in the amount of *existing* lunacy in Middlesex, without there being necessarily an increase in the amount of *occurring* lunacy. I forward you a reprint of an article which appeared in the *Journal of Mental Science*, Oct. 1886, 'On the Alleged Increase of Insanity,' which will, I hope, reassure your mind upon this point. It is quite another question whether there might not be a more economical mode of accommodating pauper lunatics than that which is being adopted in the instance to which you refer. For those who are incurable—the great majority—provision might properly be made on the moderate scale, carried out with success by the Board of the Metropolitan District Asylums. Your 'Annotation' will be of use if it leads the Justices to consider whether all has been done that ought to be done in constructing less expensive buildings for 'dements' and imbeciles, than those which are required where acute and recent cases have to be treated."

